# Elimination of *Dirofilaria immitis* Infection in Dogs, Linosa Island, Italy, 2020–2022

**DOI:** 10.3201/eid2908.221910

**Published:** 2023-08

**Authors:** Emanuele Brianti, Ettore Napoli, Giovanni De Benedetto, Luigi Venco, Jairo Alfonso Mendoza-Roldan, Angelo Basile, Marcos Antônio Bezerra-Santos, Jason Drake, Roland Schaper, Domenico Otranto

**Affiliations:** University of Messina, Messina, Italy (E. Brianti, E. Napoli, G. De Benedetto, A. Basile);; Ospedale Veterinario Città di Pavia, Pavia, Italy (L. Venco);; University of Bari, Valenzano, Italy (J.A. Mendoza-Roldan, M.A. Bezerra-Santos, D. Otranto);; Bu-Ali Sina University, Hamedan, Iran (D. Otranto);; Elanco Animal Health, Monheim, Germany (J. Drake);; RS Consultancy, Monheim (R. Schaper)

**Keywords:** *Dirofilaria immitis*, dog, elimination, heartworm disease, moxidectin, soft-kill treatment, vector-borne infections, parasites, Italy

## Abstract

On Linosa Island, Italy, *Dirofilaria immitis* infection has been hyperendemic in dogs and seroprevalent among islanders. In 2020, we implemented a heartworm disease elimination program on Linosa Island. Of 54 dogs tested for *D. immitis* antigen and microfilariae, 28 had positive results and received treatment with oral doxycycline twice daily for 4 weeks plus topical imidacloprid/moxidectin monthly for 12 months. The 26 dogs with negative results received monthly topical imidacloprid/moxidectin as preventive. During month 1, the number of microfilaremic dogs was reduced by 76.5%. From month 2 on, all animals were microfilariae negative, and during months 3 to 9, the number of antigen-positive dogs decreased progressively. Treatment of positive dogs coupled with chemoprophylaxis for noninfected dogs was effective, protecting them from new infections. The elimination program reduced the risk for human infection, representing a One Health paradigm. Monitoring and chemoprophylaxis are advocated to maintain the status of heartworm disease–free area.

*Dirofilaria immitis* and *D. repens* (Spirurida, Onchocercidae) nematodes are among the most common species of filariae that cause diseases in dogs and other animals; both species infect humans (*1*,*2*). In dogs, *D. immitis* filariae cause the severe illness heartworm disease (HWD), whereas *D. repens* infection is less severe. Both *Dirofilaria* species are transmitted by mosquitoes; in Europe, the competent vectors are *Culex pipiens* and *Aedes albopictus* mosquitoes (*3*). However, the involvement of flying insects other than mosquitos has been recently hypothesized, including black flies belonging to the *Simulium turgaicum* complex (*4*) and *Culicoides paolae* biting midges (*5*).

Unlike *D. repens* filariae, which are more widely distributed in Europe, including the Italian Peninsula (*1*,*6*–*8*), *D. immitis* worms are more frequently recorded in central Europe (*9*), including regions in northern Italy (*10*,*11*). Nevertheless, autochthonous cases of HWD in dogs from central and southern Italy have been retrospectively reported from 2009 (*1*) through 2019 (*12*); highly endemic foci in southern regions of Italy have been described (*13*,*14*). That new epidemiologic scenario developed after the arrival of a new invasive mosquito species (i.e., *Ae. albopictus*) and the increased movement of animals throughout the country combined with lack of chemoprophylactic strategies for dogs from non–HWD-endemic regions (*9*,*12*,*13*). The island of Linosa, Italy, represents a paradigm of that scenario. A highly endemic focus of *D. immitis* filariae was recently described on the small and remote island located in the middle of the Mediterranean Basin, where 58.9% of dogs tested positive for HWD by modified Knott and SNAP 4Dx Plus tests (*14*). In the same epidemiologic context, 7.9% of human islanders tested positive for *D. immitis* antibodies (*15*), which emphasizes the role of *D. immitis–*infected dogs as a source for human infections in specifically isolated environments, thus advocating for treating infected animals in the context of One Health.

Traditionally, HWD is treated with melarsomine dihydrochloride, the sole registered heartworm adulticide drug (*16*,*17*). However, an alternative therapeutic approach, known as the slow-kill protocol, that combines macrocyclic lactones with doxycycline, targeting the bacterial endosymbiont *Wolbachia*, has been used successfully in experimentally and naturally infected dogs, (*18*–*20*). That protocol was recently recognized by the American Heartworm Society and the European Society of Dirofilariosis and Angiostrongylosis as an alternative strategy when treatment with melarsomine is either unavailable or contraindicated. Compared with the melarsomine treatment, doxycycline (10 mg/kg 2×/d) for 4 weeks combined with monthly administration of a topical formulation of 10% wt/vol imidacloprid and 2.5% wt/vol moxidectin for 9 months proved to be safe and effective for treating HWD in naturally infected dogs and for clearing dogs of circulating microfilariae (*20*).

Because of its geographic and epidemiologic peculiarities, Linosa Island offered an exceptional opportunity to use this elimination program for HWD. The program involved therapeutically and prophylactically administering the alternative protocol to all infected dogs and administering monthly preventive of 10% wt/vol imidacloprid plus 2.5% wt/vol moxidectin to all remaining uninfected dogs on the island. The study was complemented by an entomologic survey to assess mosquito vectors within this unique epidemiologic context.

The study was approved by the ethics committee of animal experiments of the Department of Veterinary Medicine, University of Bari, Italy (approval number 01/2021). It was conducted according to the VICH GL9 principles of Good Clinical Practice (*21*).

## Methods

### Animal Sampling and Diagnosis

In October 2020 (T0), we physically examined all 58 dogs on Linosa Island and recorded signalment and history (e.g., age, sex, breed, clinical signs, and treatments) in individual files. At T0, we collected blood samples from each dog and stored them in two 1-mL K_3_EDTA tubes and in one 5-mL tube with clot activator. We used the Knott test (*22*) to detect and identify circulating microfilariae in whole-blood samples and a duplex real-time quantitative PCR (qPCR) to differentiate *Dirofilaria* species (*23*).

We analyzed serum samples for the presence of *D. immitis*–specific antigens by using the SNAP 4Dx Plus rapid ELISA (IDEXX, https://www.idexx.com). We considered dogs that were positive for *D. immitis*, either microscopically, serologically, or molecularly, to be infected and assigned them to the treatment group (G1); we assigned dogs that were negative to the prevention group (G2). Before the beginning of treatment, we performed cardiac ultrasonography for all *D. immitis*–infected dogs to detect adult parasites in the pulmonary arteries. We used an echocardiographic unit (Vivid-iQ; GE Healthcare, https://www.gehealthcare.com) equipped with dedicated multifrequency phased array transducers (6s-RS and M5-RS). In brief, we used the right parasternal long-axis view optimized for the right pulmonary vein and artery (standard view 1) and the right parasternal short-axis view optimized for the right pulmonary artery (standard view 2) to detect worms (*24*). We did not perform thoracic radiography because no facility was available on the island.

### Treatment and Follow-up

*D. immitis*–infected dogs (G1) received doxycycline (Ronaxan; Boehringer Ingelheim, https://www.boehringer-ingelheim.com) (10 mg/kg orally 2×/d) for 4 weeks plus a monthly application of a spot-on formulation containing 10% wt/vol imidacloprid and 2.5% wt/vol moxidectin (Advocate; Elanco Animal Health, www.elanco.com) for 12 months. Dogs in the G2 group underwent monthly chemoprophylactic treatment with the same spot-on product used for the G1 dogs.

**Figure 1:**
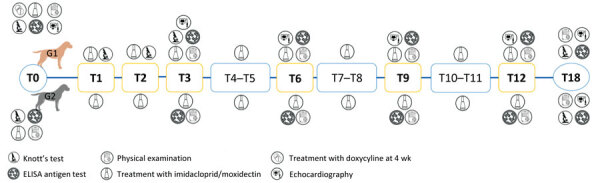
Schematic design of study of elimination of *Dirofilaria immitis* infection in dogs, Linosa Island, Italy, 2020, and follow-up examinations. G1, infected group; G2, noninjected group; T, time after start of elimination program, in months. Boldface indicates follow-up visits.

G1 dogs underwent follow-up examination at 1, 2, 3, 6, 9, 12 and 18 months (designated as T1, T2, T3, T6, T9, T12, and T18) after enrollment (Figure 1). At each follow-up visit, we clinically examined dogs and collected blood samples that we analyzed by Knott test (at T1, T2, and T18) or by SNAP 4Dx Plus test (at T1, T2, T3, T6, T9, T12 and T18). We repeated cardiac ultrasonography for G1 dogs at T3, T6, T9, T12, and T18. G2 dogs underwent clinical examination monthly, and we tested them for parasite antigenemia (by SNAP 4Dx Plus test) at T3, T6, T9, T12, and T18 (Figure 1).

Of the 58 dogs examined at T0, we included only 54 in the study because 2 dogs were going to be on the island for only a few weeks/months and the owners of the other 2 dogs did not consent to their study participation. For dogs that were permanently introduced onto the island after the beginning of the study, we performed clinical examination and specific testing (i.e., Knott test and testing for *D. immitis* circulating antigens) with owner consent and allocated them to G1 or G2 according to the test results. We advised owners to immediately report any clinical signs (i.e., cough, hemoptysis, syncope) that might appear during the study and to reduce physical activity for all infected dogs, regardless of symptoms, as much as possible.

### Mosquito Collection

**Figure 2:**
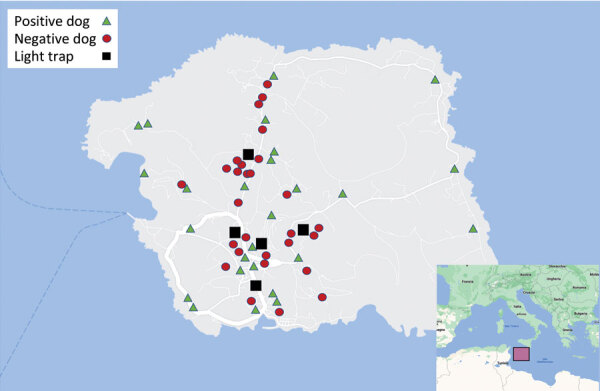
Linosa Island (Sicily, Italy) indicating the positions of infected dogs, noninfected dogs, and light traps used to capture mosquitoes. Inset shows location of Linosa Island.

We collected mosquitos from 5 locations where cases of *D. immitis* infection in dogs were detected (Figure 2). We trapped mosquitoes daily during October 2020–November 2021, 5:30 p.m.–9:00 a.m., by using 1 light trap per site, set ≈50 cm above the ground. We replaced net bags daily and stored insects in the net bags at −20°C according to collection site/day. After morphologically identifying mosquitoes to the species level (*25*), we further analyzed female mosquitoes with qPCR to detect and differentiate *Dirofilaria* spp.

### Molecular Diagnosis

We extracted genomic DNA from samples of dog blood by using the commercial QIAamp DNA Micro Kit (QIAGEN GmbH, https://www.qiagen.com) and samples of mosquito abdomen and thorax by using an in-house method (*26*). We tested all DNA samples with qPCR by using 2 species-specific primer sets targeting partial cytochrome *c* oxidase subunit 1 for *D. immitis* DNA and the second internal transcribed spacer-2 of nuclear ribosomal DNA for *D. repens* DNA, as previously described (*23*). We tested all DNA samples in duplicate and included positive and negative controls in each qPCR run.

## Results

We detected *D. immitis* infection in 28 (51.9%) of 54 (33 male, 21 female) enrolled dogs (Appendix). We found circulating microfilariae in 17 (60.7%) infected dogs and specific *D. immitis* antigens in 27 (96.4%). All microfilaremic animals except 1 had positive ELISA antigen test results (Appendix). Microfilariae were morphologically and molecularly identified as *D. immitis*. 

At the time of enrollment (T0), we conducted echocardiography on 23 of 28 infected dogs and found *D. immitis* adults within the pulmonary arteries of 20 (Appendix). On T0, we allocated 28 dogs to G1 and 26 to G2. A total of 4 dogs (3 in G1 and 1 in G2) were unavailable for follow-up analysis because of events not associated with treatment (e.g., low owner compliance or neoplastic disease). During the study period, 5 new dogs were permanently introduced onto the island (3 in January 2021 and 2 in March 2021); they tested negative for *D. immitis* infection and were included in group G2.

At T1, the number of microfilaremic dogs was reduced by 76.5%; only 4 of 17 dogs were positive by Knott test. From T2 until the end of the study, no microfilariae were found in G1 dogs. By T1, 3 infected dogs had become antigen negative (no antigen detected) according to ELISA test (Appendix). From T3 on, the number of antigen-positive dogs decreased progressively (20 at T3, 10 at T6, and 2 at T9) (Appendix). All dogs in G1 were negative for *D. immitis* circulating antigens at the 1-year follow-up visit (T12, October 2021).

Cardiac ultrasonography showed filarial parasites in the pulmonary arteries in 14 dogs at T3, 9 dogs at T6, 6 dogs at T9, and 3 dogs at T12 (Appendix). At T18, all G1 dogs scored negative by Knott test and ELISA antigen test, and no parasites were detected by echocardiography. Group 2 dogs tested negative for *D. immitis* antigens at all scheduled follow-up visits.

In this study, treatment with doxycycline and 10% wt/vol imidacloprid plus 2.5% wt/vol moxidectin seemed to be safe, and no adverse events were observed. However, in 1 dog from the G1 group, a thromboembolic-like disorder with hindlimb paralysis and pain was observed a few days after the beginning of treatment. Doxycycline treatment was temporarily suspended, and fluid therapy was promptly administered in association with unfractionated heparin (200 U/kg subcutaneously every 8 h for 3 d) and prednisone (1 mg/kg 2×/d in gradually decreasing doses for 15 d), resulting in full recovery of that G1 animal after 5 days.

Another dog that was apparently healthy at T0 exhibited ascites and diffuse edema of the hindlimbs at the clinical examination conducted on T3. Ultrasonography revealed a high parasite burden within the pulmonary arteries, associated with posthepatic portal hypertension. Treatment with sildenafil (2 mg/kg 3×/d for >6 mo) was administered. At the T6 follow-up visit, the dog had improved clinically, although pulmonary hypertension was still present, but pulmonary hypertension was absent at the next follow-up visits.

During this study, we collected 359 mosquitoes (169 male and 190 female) belonging to 6 species, the most abundant of which was *C. pipiens* (Table). Of the 190 females collected, 53 were engorged, and none of the analyzed specimens scored positive to *Dirofilaria* spp. DNA.

**Table:** Number and species of mosquitoes captured with light traps on Linosa Island, Italy, October 2020–November 2021

Mosquito species	No. (%) specimens	No. female	No. male
*Culex pipiens*	114 (31.8)	57	57
*Culiseta annulata*	69 (19.2)	38	31
*Aedes mariae complex*	55 (15.3)	37	18
*Culex laticinctus*	53 (14.8)	27	26
*Culex* spp.*	41 (11.4)	16	25
*Aedes albopictus*	16 (4.5)	8	8
*Culiseta langereolata*	6 (1.7)	5	1
*Aedes* spp.*	5 (1.4)	2	3
Total	359 (100)	190	169

*Ruined mosquito specimens were morphologically identified to genus level only.

## Discussion

In a short time, *D. immitis* parasites were eliminated from Linosa Island after we combined an alternative adulticide protocol for infected dogs with administration of preventives to noninfected animals. The peculiar epidemiologic context of the island (i.e., geographically confined area, limited number of dogs, and absence of wild reservoirs) aided the campaign. After the HWD focus in southernmost Europe was described (*14*), we planned and successfully implemented our project. In contrast to programs targeting other mosquitoborne diseases, such as malaria, this elimination campaign did not target the vector (*27*) and instead targeted the parasite specifically by eliminating the circulating juvenile forms and adult parasites in infected dogs while simultaneously protecting noninfected animals with chemoprophylaxis.

Treatment with doxycycline and imidacloprid/moxidectin was effective and safe for all of the dogs; side effects were observed in only 2 dogs, probably resulting from thromboembolism after worm death, which is a side effect associated with any adulticide protocol. Nevertheless, the risk for thromboembolism is also linked to patients’ excessive physical activity after treatment, which cannot be ruled out (*28*). Furthermore, protocols that cause the progressive, slow death of adult parasites expand the temporal window of thromboembolic risk compared with conventional treatment (*24*). A retrospective study suggests that the risk for thromboembolic events is higher 3 months after the start of the alternative treatment protocol than after start of the melarsomine protocol and that exercise restriction for treated animals is advisable for longer periods or until antigen test results are negative (*28*,*29*). Regardless, the alternative protocol has proven to be effective (*20*,*24*,*30*) and may result in fewer adverse events than those resulting from melarsomine alone (*31*,*32*) if exercise restriction and other precautions are adequately implemented.

For this study, we chose the combined doxycycline/moxidectin protocol over the conventional adulticide therapy, considering the potential adverse events associated with the sudden death of adult worms causing pulmonary thrombosis (*32*) and the lack of veterinary services available on the island. On the other hand, the adulticide effects of doxycycline on developing and adult stages of the parasite are slow (*33*), and macrocyclic lactones are effective against *D. immitis* L3 and L4 and kill only adult parasites after prolonged use (*2*). Whether the 2 drugs work better together to eliminate *D. immitis* parasites in a short time because of a cumulative or synergistic effect is not known.

Eliminating circulating microfilariae in infected dogs after the beginning of the study (T2) drastically reduced the number of dogs acting as reservoirs on the island and therefore the risk for *D. immitis* infection of mosquitoes. All of the actions contributed to breaking the life cycle of the parasite, a finding that was supported by the fact that none of the examined mosquitoes scored positive for *Dirofilaria* spp. DNA up to November 2021, unlike before the study had started a few months earlier (*14*).

The protocol that we used led to progressive reduction of detectable antigens in treated animals from T3 to T9 and to dogs testing negative at T12. This finding is similar or even better than what was recorded in previous studies that used the same protocol (*20*,*34*,*35*). Also, the conversion to the antigen-negative status observed was faster than that recorded with ivermectin alone (*24*,*36*) or with ivermectin (every 2 weeks for 6 months) and doxycycline (10 mg/kg/d orally for 30 d) in which only 73% or 80% of treated dogs reached antigen-negative status (*30*,*31*).

The use of macrocyclic lactones as adulticides has been discouraged by several authors because it may take many months for adult worms to die, enabling the disease to progress and allowing for the potential selection of macrocyclic lactone–resistant strains (*37*). Moreover, potential interference with antigen test results has also been suggested (*37*). Several studies have reported false-negative antigen test results in dogs and cats treated with macrocyclic lactones, presumably resulting from formation of immune complexes and the consequent binding of the antigens (*37*–*39*). To overcome this problem, 2 consecutive negative antigen test results 6 months apart have been suggested as a valid criterion for considering a dog cleared of infection (*34*). Therefore, in this elimination program, all dogs were probably cured of infection because in no dog was antigen detected at T12 or 6 months after (T18). This finding is also strongly supported by ultrasonography in which no adult heartworms were observed at the T18 follow-up visit. Our data may also be useful in where *D. immitis* parasites are endemic and melarsomine is not commercially available (e.g., Brazil, eastern Europe).

Despite the presence of domestic and feral cats on Linosa Island (*40*), our study did not include those hosts because of lack of owner compliance and the difficulty of sampling these animals in a remote environment. However, considering the occurrence and possible role of cats as reservoirs of *D. immitis* parasites (*41*), further studies evaluating the prevalence of this parasite in cat populations on Linosa Island are warranted.

Considering the zoonotic potential of *D. immitis* worms, elimination of HWD in dogs is pivotal for reducing the risk for human infection. Indeed, in previous surveys conducted on the human population from Linosa Island, up to 7.9% of islanders were positive for circulating *D. immitis* antibodies (*15*), which represented one of the highest percentages of human exposure ever reported. Therefore, results from our study suggest that adulticide treatment of all infected dogs, coupled with prevention for noninfected dogs, not only protects animals from HWD but may also potentially reduce the risk for human infection. Such an approach represents a paradigm for the One Health concept. Continuous monitoring and chemoprophylaxis of the canine population on the island and of all newly introduced dogs are highly recommended to maintain the status of HWD-free area.

Appendix Additional results for study of elimination of *Dirofilaria immitis* infection in dogs, Linosa Island, Italy, 2020.
