# Supplementary material for: Elimination of Dirofilaria immitis Infection in Dogs, Linosa Island, Italy, 2020–2022
Source: Emerg Infect Dis. 2023 Aug;29(8):1559–65. doi: 10.3201/eid2908.221910 (PMC10370836; doi:10.3201/eid2908.221910)
Supplement: Appendix — Additional results for study of elimination of Dirofilaria immitis infection in dogs, Linosa Island, Italy, 2020. [file 22-1910-Techapp-s1.pdf]

Article DOI: https://doi.org/10.3201/eid2908.221910

EID cannot ensure accessibility for supplementary materials supplied by authors. Readers who have difficulty accessing supplementary content should contact the authors for assistance.

Elimination of *Dirofilaria immitis* Infection in Dogs, Linosa Island, Italy, 2020–2022

Appendix

Appendix Table. Data and results of diagnostic tests performed on 28 dogs infected with *Dirofilaria immitis* and treated with doxycycline (10mg/kg BID PO for 4 weeks) combined with a monthly application of a spot-on formulation containing 10% w/v imidacloprid and 2.5% w/v moxidectin for 12 mo

			T0 October 20				T1 November 20		T2 December 20		T3 January 21			T6 April 21			T9 July 21			T12 October 21			T18 April 22			
					Echo							Echo			Echo			Echo			Echo				Echo	
ID	Sex	Age (months)	Kott (Mfl/mL)	Ant	V1	V2	Knott (Mfl/mL)	Ant	Knott (Mfl/mL)	Ant	Ant	V1	V2	Ant	V1	V2	Ant	V1	V2	Ant	V1	V2	Knott (Mfl/mL)	A	V1	V2
3	M	36	POS (902)	P	1	1	NEG	P	NEG	P	P	0	1	N	0	0	N	0	0	N	0	0	NEG	N	0	0
6	M	48	NEG	P	-	-	NEG	P	NEG	P	P	0	1	N	0	0	N	0	0	-	-	-	-	-	-	-
8	M	36	POS (1,187)	P	1	1	NEG	P	NEG	P	P	0	0	N	0	0	N	0	1	N	0	0	NEG	N	0	0
13	M	132	POS (5,311)	P	1	1	NEG	P	NEG	P	P	1	1	P	1	1	N	0	1	N	1	1	NEG	N	0	0
14	F	120	POS (227)	N	1	1	NEG	N	NEG	N	N	1	1	-	-	-	-	-	-	-	-	-	-	-	-	-
15	M	120	POS (3,876)	P	-	-	POS (36)	P	NEG	P	P	-	-	-	-	-	-	-	-	-	-	-	-	-	-	-
16	M	96	NEG	P	0	0	NEG	N	NEG	N	N	0	0	N	0	0	N	0	0	N	0	0	NEG	N	0	0
17	M	84	POS (4)	P	0	1	NEG	P	NEG	P	P	0	0	N	0	0	N	0	0	N	0	0	NEG	N	0	0
22	F	120	NEG	P	0	1	NEG	P	NEG	P	P	0	0	N	0	0	N	0	0	N	0	1	NEG	N	0	0
23	F	96	NEG	P	0	1	NEG	P	NEG	P	P	1	1	N	0	1	N	0	0	N	0	0	NEG	N	0	0
24	M	60	NEG	P	0	1	NEG	P	NEG	P	P	0	0	N	0	0	N	0	1	N	0	0	NEG	N	0	0
25	M	48	NEG	P	1	1	NEG	P	NEG	P	P	0	0	P	0	1	N	0	1	N	0	0	NEG	N	0	0
26	F	60	NEG	P	1	1	NEG	P	NEG	P	N	0	0	P	0	0	N	0	0	N	0	1	NEG	N	0	0
27	F	60	NEG	P	0	1	NEG	P	NEG	N	N	0	0	N	0	0	N	0	0	N	0	0	NEG	N	0	0
30	M	84	POS (1,147)	P	0	1	NEG	N	NEG	P	N	0	1	N	0	0	N	0	0	N	0	0	NEG	N	0	0
31	F	48	POS (7)	P	0	1	NEG	P	NEG	P	P	0	1	P	0	0	N	0	0	N	0	0	NEG	N	0	0
35	F	60	POS (1,178)	P	1	1	NEG	P	NEG	P	P	0	0	P	0	0	P	0	0	N	0	0	NEG	N	0	0
36	M	120	POS (1,141)	P	1	1	POS (2)	P	NEG	P	P	1	0	P	0	0	P	0	0	N	0	0	NEG	N	0	0
37																										

			T0 October 20				T1 November 20		T2 December 20		T3 January 21			T6 April 21			T9 July 21			T12 October 21			T18 April 22			
		Age	Kott		Echo		Knott		Knott			Echo			Echo			Echo			Echo		Knott		Echo	
ID	Sex	(months)	(Mfl/mL)	Ant	V1	V2	(Mfl/mL)	Ant	(Mfl/mL)	Ant	Ant	V1	V2	Ant	V1	V2	Ant	V1	V2	Ant	V1	V2	(Mfl/mL)	A	V1	V2
	F	108	POS (3,573)	P	1	1	NEG	N	NEG	N	N	0	0	N	0	1	N	0	1	N	0	0	NEG	N	0	0
38	F	48	POS (1,368)	P	0	0	NEG	P	NEG	P	P	1	1	N	0	0	N	0	0	N	0	0	NEG	N	0	0
39	M	24	NEG	P	0	1	NEG	P	NEG	P	N	0	1	P	0	1	N	0	0	N	0	0	NEG	N	0	0
40	M	36	NEG	P	-	-	NEG	P	NEG	P	N	0	0	P	1	0	N	0	0	N	0	0	NEG	N	0	0
43	M	96	POS (1,471)	P	0	0	NEG	P	NEG	P	P	0	0	N	0	0	N			N	0	1	NEG	N	0	0
44	M	48	POS (6,432)	P	0	1	NEG	P	NEG	P	P	0	1	N	0	0	N	0	0	N	0	0	NEG	N	0	0
45	M	134	POS (270)	P	1	1	NEG	P	NEG	P	P	1	1	N	1	1	N	0	0	N	0	0	NEG	N	0	0
49	F	134	POS (277)	P	1	1	NEG	P	NEG	P	P	1	1	P	1	0	N	0	0	N	0	0	NEG	N	0	0
53	F	156	POS (13,180)	P	-	-	POS (5)	P	NEG	P	P	0	1	P	0	1	N	0	1	N	0	0	NEG	N	0	0
55	M	156	POS (2,608)	P	-	-	POS (1)	P	NEG	P	P	0	0	N	0	0	N	0	0	N	0	0	NEG	N	0	0
	Total		17	27	20		4	24	0	24	20	14		10	9		2	6		0	3		0	0	0	0

Legend: Ant = ELISA antigen test; Echo = echography of pulmonary vein and arteries (V1) and parasternal short axis of pulmonary artery (V2).
